# The evolving role of methanogenic archaea in mammalian microbiomes

**DOI:** 10.3389/fmicb.2023.1268451

**Published:** 2023-09-01

**Authors:** James G. Volmer, Harley McRae, Mark Morrison

**Affiliations:** ^1^Centre for Microbiome Research, School of Biomedical Sciences, Queensland University of Technology (QUT), Translational Research Institute, Woolloongabba, QLD, Australia; ^2^Faculty of Medicine, University of Queensland Frazer Institute, Translational Research Institute, Woolloongabba, QLD, Australia

**Keywords:** methanogen, methanogenic archaea, methane mitigation, archaea, host-associated archaea

## Abstract

Methanogenic archaea (methanogens) represent a diverse group of microorganisms that inhabit various environmental and host-associated microbiomes. These organisms play an essential role in global carbon cycling given their ability to produce methane, a potent greenhouse gas, as a by-product of their energy production. Recent advances in culture-independent and -dependent studies have highlighted an increased prevalence of methanogens in the host-associated microbiome of diverse animal species. Moreover, there is increasing evidence that methanogens, and/or the methane they produce, may play a substantial role in human health and disease. This review addresses the expanding host-range and the emerging view of host-specific adaptations in methanogen biology and ecology, and the implications for host health and disease.

## Introduction

Methanogens are prokaryotic organisms that couple energy production and growth to the formation of methane. As such, all known methanogens are obligate methane producers, require anaerobic conditions for growth, and belong to the domain Archaea (Shalvarjian and Nayak, 2021). It is likely that methanogenesis was a dominant metabolic process around 3.5 billion years ago, with evidence suggesting it is one of the earliest mechanisms of metabolism (Lyu et al., 2018). Methanogens act as terminal electron acceptors in these anaerobic environments and are often found in habitats with a low abundance of other electron acceptors, such as sulfate (Lyu et al., 2018). Methanogens are found in a wide variety of anaerobic habitats, including wetlands, marine and freshwater sediments, soil, hot springs, landfills, rice paddy fields, and the digestive tracts of humans and other animals (Aschenbach et al., 2013; Buan, 2018). Although all currently known methanogens are obligate anaerobes, numerous species encode genes for oxygen resistance, suggesting they are capable of surviving short periods of oxygen exposure (Chibani et al., 2022). Historically, the microbial surveys of environmental microbiomes have largely accounted for methanogen diversity. However, with the intensification of high-throughput sequencing technologies applied to gut microbiome analyses, there has been an expansion in our awareness and understanding of the roles of host-associated Archaea, in particular methanogens.

## Host-associated methanogenesis

**Figure 1:**
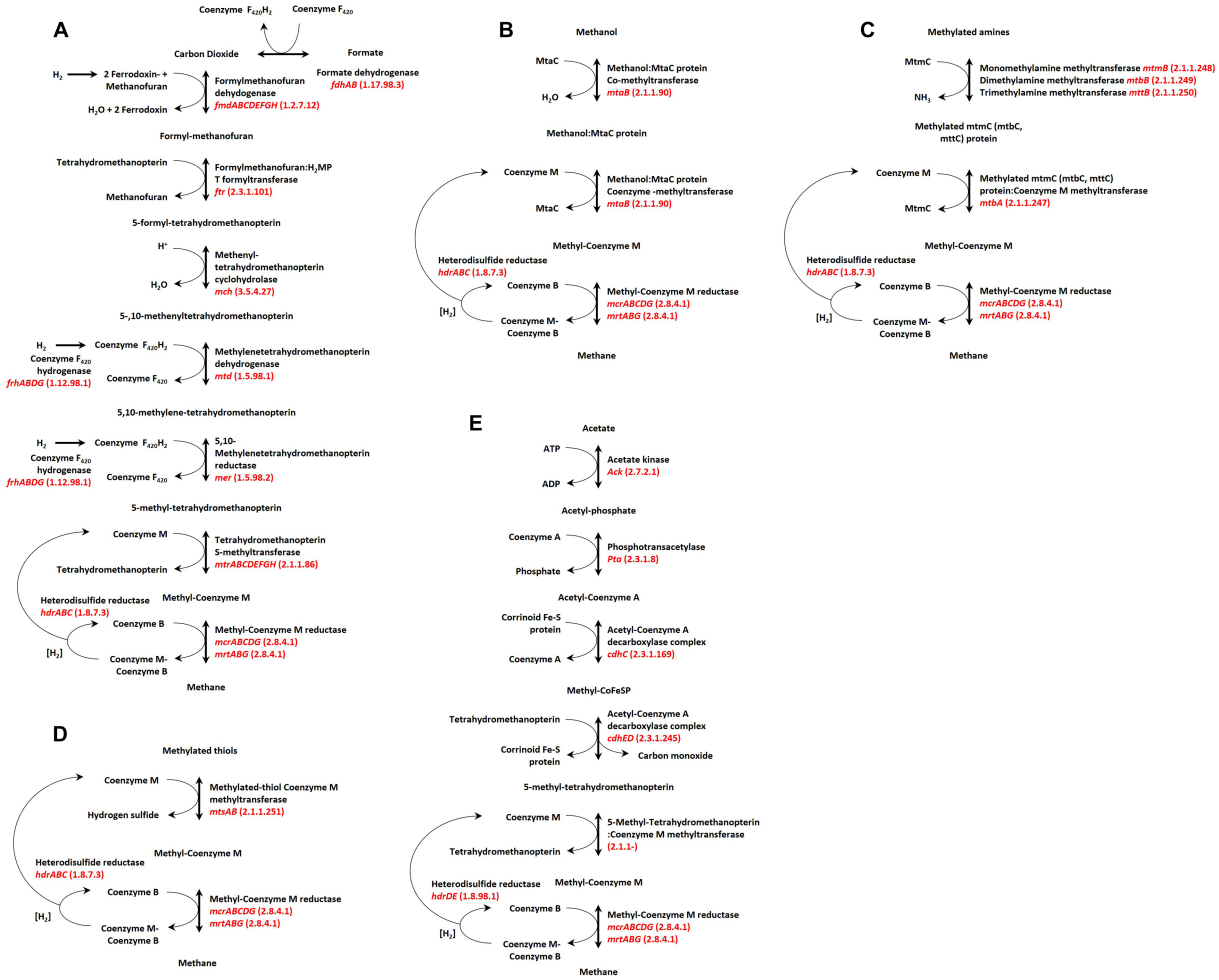
Common pathways of hydrogenotrophic, acetoclastic, and methylotrophic methanogenesis. **(A)** Acetoclastic pathway utilizing acetate, **(B)** hydrogenotrophic pathway utilizing carbon dioxide (or formate). **(C–E)** represent methylotrophic pathways for methylated thiols, methylated amines, methanol, respectively. Figure adapted from Gilmore et al. (2017), with additions based on available methanogenesis KEGG pathways (Kanehisa et al., 2016) and those described by Kurth et al. (2020).

Methanogens utilize a relatively narrow range of carbon sources, and the enzymes that catalyze their conversion to methane are often membrane-bound and coupled with ion (proton) translocation systems (Welte and Deppenmeier, 2011) that produce the electrochemical gradient for the synthesis of ATP (Pisa et al., 2007; Deppenmeier and Muller, 2008). As such, methanogenesis appears to be an obligatory step for energy production and growth of all methanogenic archaea, and the metabolic schema coordinating this process are currently separated into three broad groups: Hydrogenotrophic (Figure 1A), Methylotrophic (Figures 1B–D), and Acetoclastic (Figure 1E; Garcia et al., 2000) and are presented here in order of their current prevalence among host-associated lineages of methanogens. Hydrogenotrophic methanogenesis uses hydrogen to reduce carbon dioxide to methane, with some species additionally able to utilize formate. This form of methanogenesis is the most prevalent among characterized strains (and genomes), with the majority of Methanobacteriales, Methanomicrobiales, Methanococcales, Methanopyrales, and Methanocellales restricted to this pathway for energy production and growth. Methylotrophic methanogenesis involves the conversion of methanol, methylamines, and methylated thiols to methane. While most utilize a H_2_-dependent reduction of methylated compounds to methane (Fricke et al., 2006; Borrel et al., 2014; Sorokin et al., 2017) such as *Methanosphaera stadtmanae* DSMZ3091 and members of the order Methanomassiliicoccales; the existence of a small number of environmental isolates capable of H_2_-independent reduction of methanol to methane has long been known, and occurs in some host-associated strains (Hoedt et al., 2016). Acetoclastic methanogenesis, in which acetate is “split” during methanogenesis via the coordinated biochemistry of the acetyl-CoA decarbonyl (CdhCED) and carbon monoxide dehydrogenase (CdhAB) complex (Ferry, 1997; Welte and Deppenmeier, 2014) is currently the least common pathway encountered among characterised strains (and genomes) assigned to the order Methanosarcinales. This low abundance of host-associated acetolactic methanogens is counter to the high abundance observed in environmental samples, suggesting host physiology influences the overabundance of hydrogenotrophic methanogenesis observed in most animal gut microbiomes (Thauer et al., 2008).

Interestingly, short chain alcohols appear to play a role in the growth of at least some host-associated methanogens. For instance, Leahy et al. (2010) reported the synergistic effects of ethanol on the growth rate of *Methanobrevibacter ruminantium* during hydrogenotrophic growth. Additionally, a *Methanosphaera* spp. isolated from a Western Grey kangaroo (*Macropus fulginosus*) was shown to perform ethanol-dependent methanol reduction to methane, as well as the standard H_2_-dependent reduction observed in other *Methanosphaera* species (Hoedt et al., 2016). Similarly, a recent analysis of the bovine isolate *Methanobrevibacter boviskoreani* showed that ethanol, 1-propanol, and 1-butanol could serve as alternative electron donors for CO_2_ reduction to methane (Li et al., 2023). In contrast, while environmental isolates of *Methanocorpusculum* spp. are believed to utilize 2-propanol, butanol, and pentanol as a source of reducing power for CO_2_-dependent hydrogenotrophic methanogenesis, the recently isolated host-associated species *Methanocorpusculum vombati* and *Methanocorpusculum petauri* appear unable to utilize short chain alcohols in a similar manner (Volmer et al., 2023).

## Non-human host-associated methanogens

### Agricultural methane production and climate change

Methanogenesis is an important part of the Earth’s energy and carbon cycles, however excessive production of methane is undesirable, as it contributes to climate change. For this reason, there has been growing interest in methanogens, as methane is now known to be the second most important greenhouse gas following carbon dioxide (Lyu et al., 2018). Reducing emissions from agriculture is one area of particular importance, as livestock are the greatest contributors of anthropogenic methane, with enteric livestock methane emissions making up approximately 30% of all anthropogenically produced methane (Gilmore et al., 2017; Smith et al., 2021). Ruminant animals in particular are of significance, as they are farmed globally on larges scales and have been shown to emit more methane than some other animals, such as horses, macropodids and rabbits, even when scaled for size, feed intake, digesta retention time and gut capacity (Clauss et al., 2020). Methanogenesis also causes the loss of 6–10% of gross energy intake in ruminants and is therefore undesirable in terms of energy efficiency and feed costs (Pinares-Patiño et al., 2013). Ruminants, such as cattle, produce large amounts of methane due to the methanogenic archaea present in their gut microbiota, which consume hydrogen and other products of microbial fermentation. This process is known as interspecies hydrogen transfer and removes waste products that would otherwise limit the growth of microbial species required for fermentation in the gut (Bryant et al., 1967; Iannotti et al., 1973; Stams and Plugge, 2009). For these reasons, it is important that a deeper understanding of methanogens, especially those in high methane producing animals, is gained so that new techniques for reducing methane emissions can be developed.

Studies have shown that the amount of methane emitted by sheep is a heritable trait and that lower methane and higher methane emitting phenotypes exist (Pinares-Patiño et al., 2013; Johnson et al., 2022). Interestingly, one study found that the methanogen abundance in sheep deemed to be “high” and “low” methane emitters was similar, and increases in methane emission by certain animals appeared to be due to increases in expression of methanogenesis pathway genes, particularly those involved in hydrogenotrophic methanogenesis (Shi et al., 2014). This indicates that the composition of the gut methanogen community, rather than the total abundance of methanogens, contributed to differences in methane emissions. A similar study demonstrated that “high” and “low” methane phenotypes also exist in cattle and determined that *Methanobrevibacter* were more numerous in the “high” methane emitters, while the bacterial community in the “low” emitters included a higher abundance of Proteobacteria, in particular *Succinivibrionaceae* (Pope et al., 2011; Wallace et al., 2015). Furthermore, Martínez-Álvaro et al. (2020) found that high methane emitting ruminants were associated with a lower diversity of hydrogenotrophic methanogens, while low methane emitters were associated with an increase in methanogen species diversity across all three methanogenic pathways. *Methanomethylophilus* (a methylotrophic methanogen) was increased in lower methane emitting bovines, along with a decrease in *Methanobrevibacter* species. This study also highlighted that methane emissions in ruminants is affected by the complexity and diversity of the microbial community and the metabolism of these microbes. Together, these analyses suggests that both methanogens and other microorganisms play an important role in the different low and high methane emitting ‘ecotypes’. One such example is that rumen methanogen *Methanobrevibacter ruminantium* M1 is capable of binding to both protozoal and bacterial partners that produce hydrogen (Ng et al., 2016). Co-culture studies have also demonstrated inter-species H_2_ transfer from *Ruminococcus flavefaciens*, *Ruminococcus albus*, and *Selenomonas ruminantium* (Scheifinger et al., 1975; Latham and Wolin, 1977; Wolin et al., 1997). Bauchop and Mountfort (1981) showed that co-culture of a hydrogenotrophic methanogen with the fungi *Neocallimastix frontalis* resulted in a significant decrease in the concentrations of formate, ethanol and lactate. Methanogen have also been detected in cultures of ruminant fungi, such as *Neocallimastix* and *Anaeromyces*, though the exact fungi-methanogen interactions remain to be determined (Jin et al., 2011). Therefore, to fully understand methanogenesis, we need to learn more about these organisms and how they interact with other members of the gut microbiota.

There is evidence that the rumen microbiota is affected by the host, environment, diet and geographical location of the animal. Malik et al. (2021) investigated the effects of host species on the methanogen communities in ruminants by comparing the methane emissions and methanogen diversity of cattle and buffalo fed the same diet and kept in similar environments in the same geographical location. Cattle had higher overall methane emission levels compared to buffalo, but both species had a similar methane yield when calculated as grams of methane per dry matter intake (cattle on average had higher dry matter intake and higher body mass). There were some differences in the diversity of methanogen species present at very low abundances between the two host species, but overall, the rumen samples from cattle and buffalo had similar taxonomic profiles of their methanogens with the dominant methanogen genus being *Methanobrevibacter* for both host species. It is therefore likely that the methane yield may be more dependent on diet rather than host species for these types of hosts (Malik et al., 2021). However, cattle and buffalo are physiologically very similar and digest their food in the same way, so it may be expected that they would have similar archaeal profiles and methane yields when fed the same diet.

### Methane mitigation strategies in ruminants

Various dietary interventions have been used to attempt to reduce methane emissions from ruminants. Poulsen et al. (2013) found that supplementing dairy cattle diets with rapeseed oil reduced their methane emissions. The reduction in methane emissions was found to be the result of an inhibitory effect on the abundance and activity of a novel methylotrophic Thermoplasmata group of methanogens, as the *Methanobrevibacter* and *Methanosphaera* species also present in the rumen samples were not decreased by the addition of rapeseed oil. However, as *Methanobrevibacter* species are often the most abundant in the rumen, this may not be an effective methane mitigation strategy. Tropical tree foliage supplementation also demonstrated a reduction in methanogens and enteric methane production (Alayón-Gamboa et al., 2023). Supplementing a diet of poor-quality roughage with sweet potato vine silage has also been shown to decrease methane emissions in female cattle (Ali et al., 2019). This study found that adding sweet potato vine silage to a low-quality roughage diet increased digestibility, decreased solid digesta retention time in the rumen and decreased production of methane per unit of digested dry matter. Decreased solid retention time has been linked to decreased methane emissions in other studies, likely due to the effects of food passage time on H_2_ concentrations, and therefore methanogen activity (Janssen, 2010). Further, plant secondary metabolites have also been demonstrated as a viable anti-methanogenic supplement *ad libitum* in sheep, though the predominant *Methanobrevibacter* populations remained consistent between the test and control groups (Malik et al., 2022). Denman et al. (2007) investigated the effects of supplementing cattle diets with the anti-methanogenic chemical bromochloromethane. They found that methane emissions were reduced by around 30% in cattle supplemented with bromochloromethane compared to cattle whose feed was not supplemented. Furthermore, through the use of clone libraries generated from DNA extracted from rumen samples, they found that there was a decreased abundance of the dominant *Methanobrevibacter* species and a more diverse population of other methanogen species in cows supplemented with bromochloromethane.

One of the most successful food additives for methane mitigation to date is bromoform containing seaweed, such as *Asparagopsis* species. These seaweeds have been shown to inhibit methanogenesis in ruminants by up to 98% and are effective at low concentrations (Glasson et al., 2022). Halogenated methane analogues, such as bromoform, are proposed to inhibit methanogenesis by competitively binding with key enzymes, such as methyl coenzyme M methyltransferase. Despite its effectiveness, there are some concerns about using bromoform as a feed additive, as it is potentially carcinogenic and has ozone-depleting properties. The effects of bromoform supplementation on animal health, as well as the potential for it to enter products for consumption such as meat and milk need to be considered. Most studies to date show no increased levels of bromoform in animal products and excrement after supplementation with *Asparagopsis* (Glasson et al., 2022). However, one study by Muizelaar et al. (2021) showed increased levels of bromoform in milk and urine from cattle in the early stages of *Asparagopsis* supplementation, but these levels were still below the recommended World Health Organization limit for bromoform. This study had to be terminated early, as many of the cows refused the food mix supplemented with seaweed and therefore had low food intakes. This may indicate that the supplementation of seaweed into the diet of cattle needs to be further optimized. Another potential risk of using bromoform containing seaweed as a supplement is the highly volatile nature of this compound. Bromoform can be converted into inorganic bromine in the atmosphere, which has ozone depleting properties (Glasson et al., 2022). For bromoform to be a viable methane mitigation strategy, the environmental impacts of *Asparagopsis* production on the atmosphere, as well as land use, storage and transportation need to be considered. As the cattle industry is so large in countries like Australia, it may not be feasible to grow enough *Asparagopsis* to supplement the feed of every ruminant raised for agricultural purposes.

Another promising methane reduction strategy is the use of 3-Nitrooxypropanol (3-NOP). This molecule specifically targets and inactivates methyl-coenzyme M reductase (MCR), which is essential in catalyzing the final step of methanogenesis (Figure 1; Duin et al., 2016). In the last decade, numerous studies have shown the efficacy of 3-NOP in reducing enteric methane production, with average predicted reductions of 30% (Kim H. et al., 2020). In fact, some studies have reported the use of 3-NOP as a feed additive to reduce methane production by up to 82% (Vyas et al., 2016; McGinn et al., 2019). A recent analysis by Araújo et al. (2023) showed a reduction in methane emissions by ~49.3% (g/d) resulting in a reduction in gross energy intake loss by 42.5%. The methane mitigation potential of 3-NOP was also demonstrated with feedlot cattle fed a barley-based diet with canola oil and showed 65.5 to 87.6% reduction in emissions (Almeida et al., 2023). Several studies have also shown that 3-NOP poses no mutagenic or genotoxic potential and that Bovaer^®^ 10, a food additive containing 3-NOP, was efficacious for methane reduction in dairy cows (Thiel et al., 2019; Bampidis et al., 2021). It was further shown that the supplement did not affect soil health and 3-NOP manure could be used as a nutrient source for forage crops (Owens et al., 2021). This positions 3-NOP as a promising feed additive for reducing methane emissions whist posing minimal impacts on the animal or surrounding environment.

Li et al. (2022) showed that altering the diet of cattle changed the microbial composition of the rumen and subsequently influenced methanogenesis. Their study showed that feeding a fiber-rich versus starch-rich diet resulted in two distinct microbiomes with differing carbohydrate degradation, H_2_ metabolism and methane production. The difference in the microbiome between the two diet types was associated with distinct substrate preferences and metabolic pathways of certain microbial species. The fiber-rich diet was found to increase the acetate to propionate ratio and selected for fibrolytic bacteria. The enrichment of fibrolytic bacteria can play a role in the adaptation to lignified diets, however it may also be associated with increased methane production and a decrease in energy conversion efficiency (Li et al., 2022). The starch-rich diet increased ruminal dissolved H_2_ levels, decreased CH_4_ production and enriched for amylolytic bacteria. The fiber-rich diet enriched for methanogenic hydrogenases from Methanobacteriota species, whereas the starch-rich diet enriched for hydrogenases from Firmicutes and Spirochaetota groups. These results show that modifying cattle diets to contain more starch-rich ingredients may help mitigate methane production. Interestingly, Li et al. (2022) also found that the marker gene for hydrogenotrophic acetogenesis was more abundant in cattle fed the fiber-rich diet. This is interesting because another method that has been proposed to reduce methane production is to enrich for acetogenic bacteria in the rumen, which compete for H_2_ and produce acetate rather than methane (Karekar et al., 2022). So far, acetogenic bacteria isolated from the rumen have not been able to outcompete methanogens in *in vitro* studies under normal circumstances, however there is evidence that increased H_2_ levels, may allow homo-acetogens to become more dominant hydrogen sinks in the gut environment (Karekar et al., 2022).

Defaunation (the removal of protozoa) has also been proposed as a potential method for reducing the methanogen population in the rumen and promoting acetogenesis. These protozoa can act as hosts for methanogens and protect them while providing a source of hydrogen. *In vivo* studies suggest that these protozoa are not essential to host animal health and defaunation has shown decreases in methane production of up to 49% in animals fed barley-based concentrates (Whitelaw et al., 1984). The direct feeding of reductive acetogens to ruminant livestock has so far shown only temporary success. In one study, methane emissions were reduced by up to 80% in rams supplemented with *Peptostreptococcus productus* (a reductive acetogen), before increasing to normal levels after less than a week (Nollet et al., 1998). This suggests that the acetogenic bacteria present in the rumen were not robust enough to take over and remain as the primary H_2_ sinks (Karekar et al., 2022). An increased understanding of how these microbes compete with methanogens in the rumen may provide insight into techniques that could enrich for acetogens and other hydrogenotrophic bacteria, so that they could outcompete methanogens and mitigate methane production from cattle and other ruminants.

To date there have been a number of studies investigating various methods to mitigate methane emissions from ruminant livestock, including the introduction of microorganisms into the rumen that would compete with methanogens for hydrogen (such as reductive acetogens), the elimination of protozoa in the rumen that form symbiotic relationships with methanogens, immunization of the host against methanogenic archaea, as well as dietary supplements and additives (Goopy, 2019). However, application of these techniques to alter the host microbiome or eradicate methanogens from the rumen have often shown limited success *in vivo* (Goopy, 2019) and no single methane mitigation technique has thus far been successfully implemented on a large scale in the agricultural industry. Methane production is a heritable trait (Pinares-Patiño et al., 2013), suggesting that it is possible to produce animals with a ‘low methane phenotype’ through selective breeding. However, this process would require a major shift in the agricultural industry that would be costly and time consuming, as identifying which animals are low methane emitters requires complex testing and it would take years of selective breeding to achieve the desired phenotype. Finally, vaccination against methanogens has repeatedly been suggested as a possible solution to enteric methane emissions (Baca-González et al., 2020), but this would require a vaccine that universally targets all methanogens, so that another methanogenic species does not expand to fill the niche. Clearly, the most likely way to produce such a vaccine requires better understanding of the genetics and molecular biology of host adaptation to identify those epitopes (targets) that generate a strong, multivalent and host-specific antibody response.

### Methanogens and Australian herbivores

As well as ruminants, methanogens inhabit the gastrointestinal tract of Australian herbivores. The digestive anatomy of the rumen has some similarities, but differs to that of Australian marsupials, which can be classed as either foregut fermenters (Macropodidae) or hindgut fermenters (wombats, koalas, possums and gliders) (Hume, 1984). The gut microbiome also differs between ruminants and marsupials. However, the methanogens present in some macropodids have a similar taxonomic profile to those in ruminants, although present at substantially lower numbers (Evans et al., 2009). Despite the presence of methanogens in their digestive tract, macropodids have been shown to produce less methane than ruminants when fed the same diet (Madsen and Bertelsen, 2012). For this reason, investigating the diversity and metabolism of methanogens present in the digestive tracts of native Australian herbivores may provide insights into why these animals are ‘low’ methane emitters and provide approaches for the potential reduction of methane emissions in ruminants.

To date, there have been few studies aiming to characterize the methanogens present in Australian herbivores and determine why these animals are low methane emitters, and because of this, there are limited cultured isolates of marsupial associated methanogens. However, Evans (2011) isolated a *Methanobrevibacter* species (WBY1) from the forestomach digesta of a Tammar wallaby and obtained an enriched culture of a Thermoplasmatales (Methanomassiliicoccales) affiliated methanogen from the forestomach digesta of a Western Grey Kangaroo. WBY1 was found to grow only in the presence of CO_2_/H_2_ and the Methanomassiliicoccales-associated methanogen was found to utilize methylamines and H_2_ for methanogenesis, but an axenic culture was not achieved, which indicates that it may have relied on the bacteria present in the enrichment culture for specific metabolites. Subsequently, Hoedt et al. (2016) isolated a *Methanosphaera* species (sp. WGK6) from the digestive tract of a Western Grey Kangaroo that is capable of using ethanol and methanol, rather than H_2_ and methanol for methylotrophic methanogenesis. The proposed mechanism of this ethanol/methanol methanogenesis is a two-step oxidation of ethanol to acetate coupled with the reduction of methanol to methane. This mode of metabolism suggests that some *Methanosphaera* species have adapted to lower H_2_ environments and may be associated with lower methane emissions. Similar findings were recently confirmed in *Methanobrevibacter*, with Li et al. (2023) demonstrating that *Methanobrevibacter boviskoreani*, a rumen methanogen isolate, was capable of utilizing ethanol, 1-propanol, and 1-butanol as alternative electron donors in CO_2_-dependent methanogenesis. In a further study by Hoedt et al. (2018), a metagenomic analysis was used to compare *Methanosphaera* strains from different hosts and, interestingly, it was discovered that two genotypes exist. A larger (~2.9 Mbp) genotype was present in ruminant hosts and a smaller (~1.7 Mbp) genotype present in monogastric hosts, such as macropodids. The results of these findings demonstrate that the *Methanosphaera* genus is monophyletic and comprised of two genotypes, the larger of which is so far restricted to ruminant hosts. This demonstrates that *Methanosphaera* species have adapted to live in their specific host environments and their genome content reflects this. Recently, Volmer et al. (2023) successfully isolated two novel *Methanocorpusculum* species, *M. petauri*, and *M. vombati* from mahogany glider and wombat fecal samples, respectively. The *Methanocorpusculum* genomes were larger than those of *Methanocorpusculum* species found in environmental samples and showed a distinct phylogenetic separation from the environmental-associated genomes. The two novel *Methanocorpusculum* genomes encoded similar genes for methanogenesis, however, both were unable to utilize secondary alcohols in CO_2_-dependent methanogenesis, like the environmental isolate *M. parvum*.

There is evidence that macropodids may produce less methane than ruminants due to an increased presence of acetogens that consume more of the H_2_ produced in the kangaroo forestomach than in the rumen. Various studies, such as that by Ouwerkerk et al. (2009), have demonstrated the presence of acetogens in the macropodid forestomach. Godwin et al. (2014) used stable isotope probing to investigate the fate of H_2_ and CO_2_ in the kangaroo forestomach and the rumen. They performed *in vitro* fermentations using ^13^C labeled bicarbonate and CO_2_ with kangaroo forestomach and bovine rumen contents, in which the methane content in the headspace was measured at various intervals. They found that methane was detectable in the headspace of the rumen fermentation after only 3 h, whereas it took 7 days before a measurable quantity of methane was detected in the kangaroo fermentations. The methane produced in the kangaroo fermentations was also mostly unlabeled, which suggests that it did not originate from the CO_2_. This indicates that the methanogens present in the kangaroo forestomach may be less active than those in the rumen and also use a pathway other than hydrogenotrophic methanogenesis. Godwin et al. (2014) also found that kangaroo fermentations with ^13^C labelled bicarbonate produced highly labelled acetate, whereas the bovine fermentations produced only slightly labelled acetate. This demonstrates that reductive acetogenesis produces a larger amount of acetate in the kangaroo forestomach than the rumen. In addition to the *in vitro* fermentations, Godwin et al. (2014) used RNA stable isotope probing to identify bacterial species associated with CO_2_ and H_2_ metabolism in the kangaroo forestomach samples. They identified an OTU that is very close to the 16S sequence of *Blautia coccoides*, which is a known acetogen. Sequences with high similarity to this OTU were found in all the kangaroo samples, but only 40% of the rumen samples. One reason that acetogens are more dominant in the macropodid forestomach may be the shorter retention time of fiber when compared to the rumen (Karekar et al., 2022). Alternatively, Leng (2018) hypothesized that the morphology of the macropodid forestomach limits expansion and makes it more sensitive to high gas concentrations, which has led to selective pressure against methanogens through the excretion of immunogenic components by mucosal tissue in the cranial blind sac of the forestomach. Thus, leaving more opportunity for acetogens to fill the niche of H_2_ consumption without producing large amounts of gas. This hypothesis was based on the similarity of the cranial blind sac to other organ structures found in mammals, such as the appendix, which has been proposed to play a role in immune function and biofilm formation (Randal Bollinger et al., 2007). However, this theory proposed by Leng (2018) is yet to be backed up by experimental data. Overall, these results indicate that acetogenesis may play a larger role in CO_2_ and H_2_ metabolism in macropodids than in cattle, resulting in reduced methane emissions. However, the conditions that allow this less thermodynamically favorable pathway to operate in the macropodid forestomach are still not well understood. A better understanding of the methanogenic pathways utilized by methanogens in macropodids and their competition with other hydrogenotrophic bacteria may help to elucidate reduced methane production in ruminants.

### Methanogens in non-ruminant animals

Non-ruminant herbivores are also dependent on the recruitment and retention of microbes within different segments of their gastrointestinal tract in support of plant biomass conversion to nutrients. These adaptations to herbivory are outlined in detail by Mackie et al. (2000) but in brief detail, these animals may utilize either a sacciform, non-gastric region of the stomach (e.g., the macropodids, such as kangaroos and wallabies) or hindgut (caecum or colon) (White and Mackie, 1997). Many breeds of pigs including Duroc, Landrace, Yorkshire (Mao et al., 2011; Mi et al., 2019), and Erhualian (Zhu et al., 2011) also contain a substantial abundance of *Methanobrevibacter*. One study on Canadian pigs showed *Methanoculleus* spp. as additional major contributors to methane emissions through hydrogenotrophic methanogenesis (Barret et al., 2013). Colonic fermenters such as horses have shown an abundance of *Methanobrevibacter* (Lin and Miller, 1998; Fernandes et al., 2014) and *Methanocorpusculum* species (O'Donnell et al., 2013; Fernandes et al., 2014). Similarly, white and black rhinoceros are colonized by *Methanobrevibacter* and *Methanocorpusculum*, as well as *Methanosphaera* and *Methanomassiliicoccales-*related species (Luo et al., 2013; Gibson et al., 2019). In fact, *Methanocorpusculum* spp. was the most abundant methanogen in captive white rhinos at ~60% (Luo et al., 2013) and has also been found as the predominant taxon in Japanese thoroughbred horses and ponies (Lwin and Matsui, 2014). Smaller caecal fermenting animals also show methanogen colonization. *Methanobrevibacter*-related species have been isolated from rodents such as the feces of rats, with aging rats showing an increased abundance of total methanogens (Maczulak et al., 1989; Lin and Miller, 1998). Additionally, squirrels also contain *Methanosphaera* (Carey et al., 2013). The caecal contents of rabbits have also shown the presence of *Methanosphaera* and several *Methanobrevibacter* species (Kusar and Avgustin, 2010). *Methanosphaera cuniculi* was isolated in pure culture from the intestinal tract of a rabbit, providing genomic insights into the limited number of characterised *Methanosphaera*. The diet of the North American beaver consists entirely of woods, roots and aquatic plants, requiring a syntrophic relationship with fermentative bacteria to aid in digestion (Kohl et al., 2014). Interestingly, *Methanosphaera, Methanobrevibacter,* and *Thermoplasmatales* were detected from the caecum and feces, but *Methanosphaera-*associated OTUs accounted for more than 99% of archaeal reads (Kohl et al., 2014). Multiple sequences of *Methanosphaera* were detected across all samples, with a single OTU accounting for 85–90% of *Methanosphaera* sequences (Kohl et al., 2014). This shift toward *Methanosphaera* is likely driven by the production of methanol by bacterial fermentation of pectin derived from the plant-rich diet (Pieper et al., 1980; Revilla and González-SanJosé, 1998).

## Human-associated methanogens

### The methanogen expansion via human gut microbiome research

**Figure 2:**
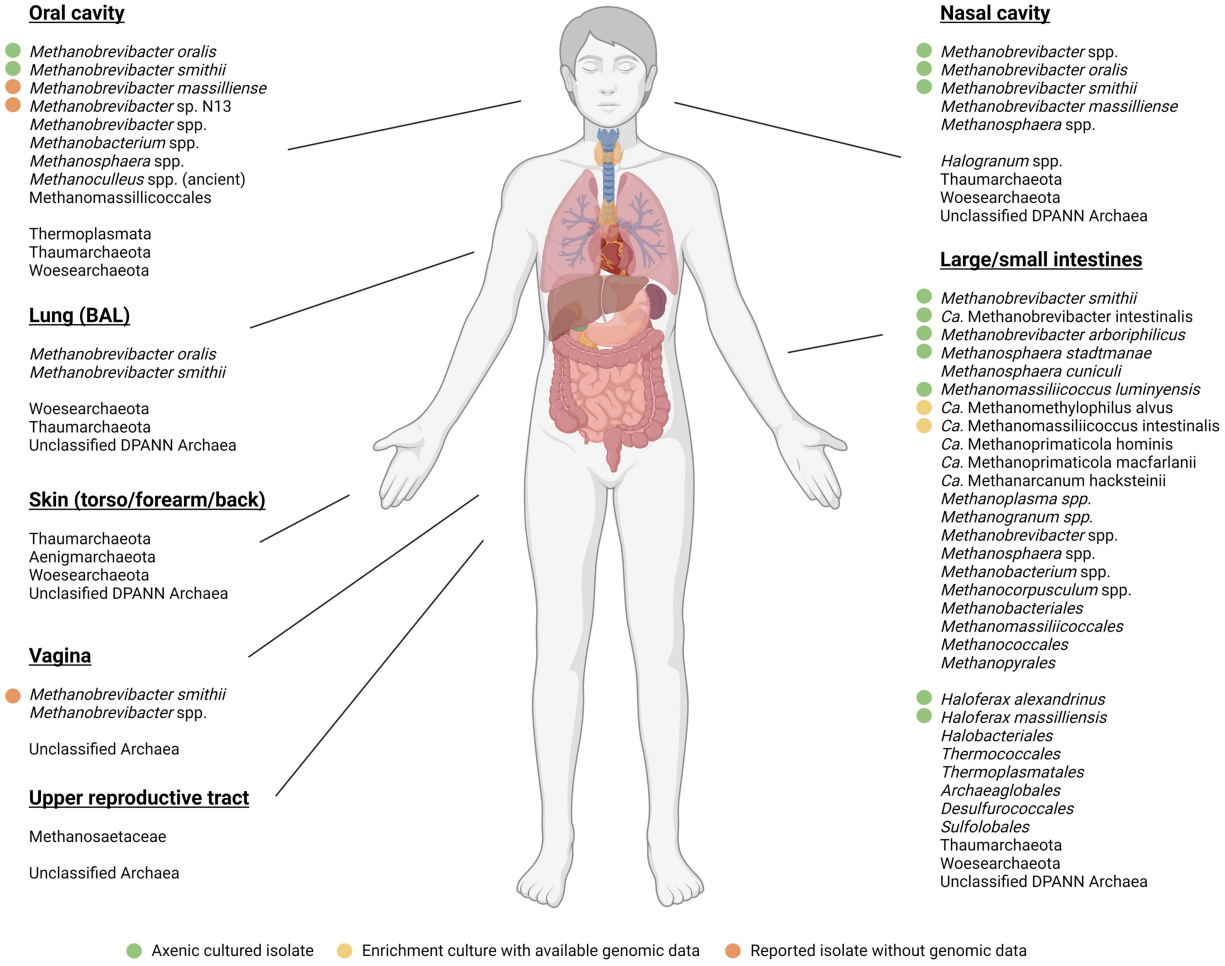
Methanogenic and other archaea detected across the human body. Samples included those from the oral cavity, nasal cavity, lungs (BAL), skin (torso/forearm/back), large/small intestine, vagina, and upper reproductive tract. 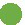 represents axenic isolates with available genomic data, 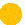 represents non-axenic enrichments with available genomic data, 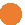 represents isolates with no available genomic data. No symbol represents identification through sequencing data alone. The Figure is adapted and augmented from Bang and Schmitz (2015) and Nkamga et al. (2017), respectively; and the anatomical diagram created with BioRender.com.

Figure 2 provides an overview of the prevailing evidence that the Domain Archaea, and the methanogens particularly are part of the human microbiome. That human methanogens are members of the human gut microbiota was first established principally by the efforts of Wolin and colleagues in the 1980s, including their isolation of the type strains of *Methanobrevibacter smithii* and *Methanosphaera stadtmanae* (Miller et al., 1982; Miller and Wolin, 1985), the latter of which prevailed for many years as the sole cultured member of the genus. More recently, the Methanomassiliicoccales (*Methanomassiliicoccales* and *Methanomethylophilus*; Figure 2) were confirmed to be present and capable of the utilization of methanol and other methylated compounds (Chaudhary et al., 2015). As such, the human-associated methanogens are similar to those isolated from other vertebrate animals in terms of their carbon utilization profile, but to date the metabolic capacity for methane formation and growth appears to be hydrogen-dependent.

*Methanobrevibacter smithii* typically represents the most dominant methanogen in the GI tract, with prevalence of nearly 95% (Dridi et al., 2009, 2011b; Dridi, 2012) and relative abundance of up to 10% in some studies (Eckburg et al., 2005). *Methanosphaera stadtmanae* are abundant and prevalent in ~30% of individuals (Dridi et al., 2012b). The *Methanomassiliicoccales,* represented by *Methanomassiliicoccus luminyensis,* are the least common, with a prevalence of 4–50% of individuals tested and increase in relative abundance with age (Dridi et al., 2012b; Vanderhaeghen et al., 2015). The use of culture-independent methods identified *Candidatus* Methanomethylophilus alvus (Borrel et al., 2012) and *Candidatus* Methanomassiliicoccus intestinalis (Borrel et al., 2013) as additional methanogens in the human gut. Methanogens have also been identified among the communities of other body sites, with *Methanobrevibacter oralis* and *Methanomassiliicoccales* spp. detected in the oral cavity (Li et al., 2009; Horz et al., 2012). Although phylogenetically similar, *M. oralis* and *M. smithii* show adaptations to their respective biological niches, with *M. oralis* isolates lacking the capacity to utilize formate (Ferrari et al., 1994) and *M. smithii* encoding for bile salt hydrolase (*bsh*) genes (Gaci et al., 2014). Other members of the *Methanobrevibacter* genus found in humans include *M. arboriphilus* (Khelaifia et al., 2014) and *M. massiliense* (Huynh et al., 2017), though there is currently little information about their respective prevalence or abundance. Interestingly, cultivation of oral methanogens from three individuals with severe periodontitis also identified a novel *Methanobrevibacter* species designated N13 (Huynh et al., 2015). Recently, Chibani et al. (2022) and colleagues, produced a comprehensive catalog of genomes recovered from human gut metagenomes. This analysis identified almost 100 archaeal strains (99% ANI), the majority of which were members of Methanobacteriales (87.15%), as would be expected. Interestingly, genomes were also recovered from Methanomicrobiales (0.26%) and Halobacteriales (0.17%), which are scarcely reported as a part of the human gut microbiome. Despite *M. luminyensis* originally isolated from a human fecal sample (Dridi et al., 2012a), no representatives of this species were recovered. In fact, *Ca.* Methanoprimaticola hominis (originally Methanomassiliicoccales Mx06 or UBA71), *Ca.* Methanomethylophilus alvus, and *Ca.* Methanomassiliicoccus intestinalis represented the most abundant species of Methanomassiliicoccales. Further, it was also identified that *M. smithii* represents two distinct species, *M. smithii* and *Ca.* M. intestinii, with distinct genes encoding molybdate transport and adhesin-like proteins, as well as additional uncharacterised processes.

Representatives of the *Methanobacteriales, Methanomicrobiales, Methanococcales, Methanopyrales,* and *Methanosarcinales* have also been detected in the human gut via shotgun metagenomics sequencing (Scanlan et al., 2008; Bang and Schmitz, 2015). Along with the abovementioned *M. arboriphilus,* methyl coenzyme M reductase A (*mcrA*) clone sequences closely related to *Methanoculleus chikugoensis* were found, along with oral representatives of *Methanobacterium congolense* and *M. mazei* (Nava et al., 2012; Nguyen-Hieu et al., 2013). A separate study on longitudinal GIT biopsies retrieved *Methanobrevibacter* sequences related to *M. filiformis* and *M. woesei*, along with the first identification of *Methanobacterium* sequences within the ileum (Koskinen et al., 2017). This study additionally showed *Methanobacteriaceae* present within nasal samples. Methanogenic archaea identified as *M. smithii* have been detected in vaginal samples of individuals suffering from bacterial vaginosis (Belay et al., 1990; Grine et al., 2019a,b). Further, a study into the reproductive tract showed *Methanosaetaceae* within cervical mucus and peritoneal fluid samples (Li et al., 2018).

**Figure 3:**
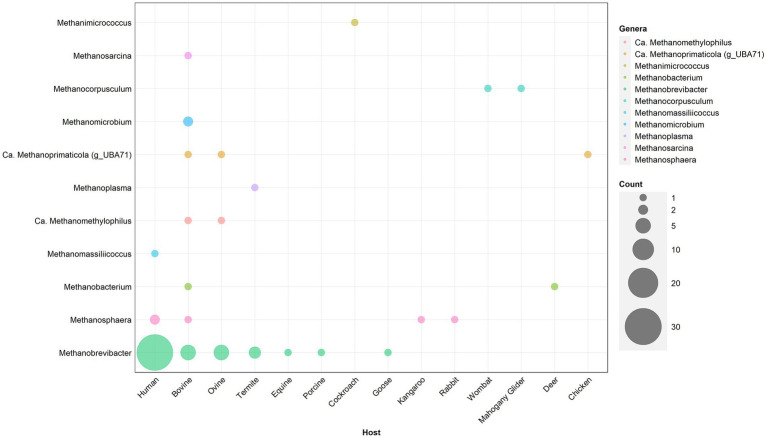
Taxonomic distribution of published methanogen isolates cultured from animal hosts. Only isolates which have been published with available whole genome sequencing have been included. The size of individual circles represents the number of isolates, with the color representing the respective animal from which the isolate was recovered. Isolates have been recovered from Methanobacteriales (*Methanobrevibacter, Methanosphaera,* and *Methanobacterium*), Methanomassiliicoccales (*Methanomassiliicoccus, Methanomethylophilus, Methanoplasma,* and *Methanoprimaticola*) (g_UBA71), Methanomicrobiales (*Methanomicrobium* and *Methanocorpusculum*), and Methanosarcinales (*Methanimicrococcus* and *Methanosarcinales*).

Given the diversity of human-associated methanogens identified by culture-independent techniques, it is important to perform functional analyses of cultured isolates to validate culture-independent findings. Current human methanogen isolates have a substantial bias toward *M. smithii*, which may be expected given the high prevalence and abundance of this species (Figure 3). Despite Chibani et al. (2022) identifying at least 16 species of GIT-associated methanogens, the vast majority have no axenic cultured representative including *Ca.* Methanoprimaticola, one of the most prevalent genera in the human gut. This bias toward *Methanobrevibacter* species is consistent across other mammalian hosts, with isolates from bovine, ovine and termite samples dominated by the genus (Figure 3). To understand the role of methanogens in human health, it is important to cultivate representatives from diverse species. Further, ~50% of all current methanogen isolates are human *M. smithii* and, as such, methanogen cultivation should also focus on recovering a variety of species from diverse animal hosts to provide phylogenetic and functional analyses on the wider role of host-associated methanogens.

In summary then, and similar to other vertebrate hosts, methanogens (and the Domain Archaea) are a relatively small population of microbes that reside within the microbiomes resident at different sites throughout the human body. There has been a gradual but sustained increasing interest in Archaea, and specifically methanogens, and their relationship with human health and disease. In that context, Table 1 summarizes the associations between the relative and/or absolute abundance of methanogenic archaea with different non-communicable diseases and disorders. For the reasons outlined above, much of the interest has been directed toward digestive health and disease, and despite these associations, studies that dissect cause from consequence in these associations with the organic and/or functional diseases and disorders remain limited.

**Table 1:** Associations between methanogenic archaea and different diseases or disorders.

Pathology	Association	Method	Citation
IBD - CD	↑ *Methanosphaera* ↑ Msp-specific IgG	RT-qPCR (*MtaB1*) Indirect ELISA	Blais Lecours et al. (2014)
	↓ *Methanobrevibacter*	Metagenomic shotgun sequencing	Lo Sasso et al. (2020)
	↓ *Methanobrevibacter*	16S rRNA Sequencing	Pascal et al. (2017)
IBD - CD/UC	↓ Methane production	Breath-methane test	Peled et al. (1987)
	↓ Methane production	Breath-methane test	McKay et al. (1985)
	↓ Methanogens	PCR (*mcrA*)	Scanlan et al. (2008)
	↑ *Methanobrevibacter*	16S rRNA RT-qPCR	Verma et al. (2010)
	↓ *Methanobrevibacter* ↑ *Methanobrevibacter* in remission	16S rRNA RT-qPCR	Ghavami et al. (2018)
	↓ Methane Metabolism	16S rRNA PICRUSt predictions	Nishino et al. (2018)
	↓ *Methanobrevibacter*	RT-qPCR	Heidarian et al. (2019)
	↓ Methane production	Breath-methane test	Gu et al. (2020)
IBS	No sig. Association	Breath-methane test	Bratten et al. (2008)
	No sig. Association	PCR (*mcrA*)	Scanlan et al. (2008)
IBS - Constipation	↑ Methane production ↑ Methane with severity	Breath-methane test	Pimentel et al. (2003)
	↑ Methane production ↑ *M. smithii*	Breath-methane test 16S rRNA RT-qPCR	Ghoshal et al. (2016)
	↑ Transit time	Breath-methane test	Attaluri et al. (2010)
	↑ Transit time	Breath-methane test	Lee et al. (2013)
	↑ Transit time	Breath-methane test	Pimentel et al. (2006)
	↑ Transit time	Breath-methane test	Majewski and McCallum (2007)
	↑ Transit time	Breath-methane test	Hwang et al. (2010)
	↑ Transit time	Breath-methane test	Ghoshal et al. (2011)
	↑ Methane with severity	Breath-methane test	Chatterjee et al. (2007)
	↑ *Methanobrevibacter*	16S rRNA Sequencing	Pozuelo et al. (2015)
	↑ *M. smithii* ↑ Methane production	16S rRNA Sequencing Breath-methane test	Kim et al. (2012)
IBS – Diarrhea	↓ Methane production	Breath-methane test	Pimentel et al. (2003)
	↓ *Methanobacteriales*	16S rRNA RT-qPCR	Tap et al. (2017)
Diverticulosis	↑ Methane production ↑ Methanogen abundance	Breath-methane test Culture-based	Weaver et al. (1986)
Colorectal Cancer	↑ Methane with severity	Breath-methane test	Pique et al. (1984)
	↑ Methane production	Breath-methane test	Haines et al. (1977)
	↓ Methanogen abundance	Breath-methane test	Segal et al. (1988)
	↓ Methanogen abundance	Metagenomic shotgun sequencing	Coker et al. (2020)
	↑ *Methanobacteriales* ↑ *Methanobrevibacter*	16S rRNA qPCR	Mira-Pascual et al. (2015)
	No sig. Association	Breath-methane test	O'Keefe et al. (2007)
	No sig. Association	Breath-methane test	Segal et al. (1988)
	No sig. Association	Breath-methane test	Karlin et al. (1982)
	No sig. Association	Breath-methane test	Kashtan et al. (1989)
	No sig. Association	Breath-methane test	Hoff et al. (1986)
	No sig. Association	PCR (*mcrA*)	Scanlan et al. (2008)
Obesity	↑ Methanogen abundance	16S rRNA Pyrosequencing	Zhang et al. (2009)
	↓ *M. smithii*	16S rRNA RT-qPCR	Million et al. (2012)
	↓ *M. smithii*	16S rRNA RT-qPCR	Million et al. (2013)
	↓ *M. smithii* ↑ Unclassified *Methanobrevibacter*	Metagenomic shotgun sequencing	Maya-Lucas et al. (2018)
	↑ Methane production	Breath-methane test	Mathur et al. (2013)
	No sig. Association	16S rRNA RT-qPCR	Schwiertz et al. (2010)
	No sig. Association	Breath-methane test 16S rRNA RT-qPCR	Fernandes et al. (2013)
Anorexia	↑ *M. smithii*	16S rRNA RT-qPCR	Armougom et al. (2009)
	↑ *M. smithii*	RT-qPCR	Million et al. (2013)
	↑ *M. smithii*	16S rRNA RT-qPCR	Borgo et al. (2017)
	↑ *Methanobrevibacter*	16S rRNA Sequencing	Mack et al. (2016)
	↑ *M. smithii*	16S rRNA Sequencing	Prochazkova et al. (2021)
Malnutrition	↓ *M. smithii*	Archaeal-specific qPCR	Million et al. (2016)
	↓ *M. smithii*	16S rRNA RT-qPCR	Camara et al. (2021)
	↑ *M. smithii*	16S rRNA Sequencing	Kamil et al. (2021)
Encopresis	↑ Methane production	Breath-methane test	Fiedorek et al. (1990)
SBS	↓ *M. smithii*	16S rRNA RT-qPCR	Boccia et al. (2017)
Mets	↑ *Methanobrevibacter*	16S rRNA Sequencing	Lim et al. (2017)
Multiple Sclerosis	↑ *Methanobrevibacter* ↑ Breath methane	16S rRNA Sequencing Breath-methane test	Jangi et al. (2016)
	↑ *Methanobrevibacter*	16S rRNA Sequencing	Tremlett et al. (2016b)
	↑ *Methanobacteriaceae*	16S rRNA Sequencing	Jhangi et al. (2014)
Parkinson’s Disease	↑ Methanogen abundance	16S rRNA Sequencing	Qian et al. (2018)
	↑ Methanogen abundance	Metagenomic shotgun sequencing	Bedarf et al. (2017)
SIBO	↑ Breath methane	Breath-methane test	Suri et al. (2018)
	↑ Breath methane	Breath-methane test	Pimentel et al. (2020)
Vaginosis	↑ *M. smithii*	Antigenic fingerprinting	Belay et al. (1990)
	↑ *M. smithii*	16S rRNA sequencing	Grine et al. (2019a,b)
Urinary Tract Infection	↑ *M. smithii*	16S rRNA/McrA PCR RT-qPCR/Culture	Grine et al. (2019a,b)
Anerobic Abscesses	M. smithii*	16S rRNA/McrA Sequencing	Nkamga et al. (2016)
	↑ *M. oralis*	16S rRNA RT-qPCR/ Metagenomic sequencing	Drancourt et al. (2017)
	M. oralis*	16S rRNA/McrA Sequencing	Nkamga et al. (2018)
Periodontal Disease	↑ *M. oralis* ↑ *M.* sp. strain N13	16S rRNA/McrA Sequencing/Culture	Huynh et al. (2015)
	M. massiliense*	Culture	Huynh et al. (2017)
	↑ *M. oralis*	Archaeal PCR	Li et al. (2009)
Parasitic Infection	↑ *Methanobrevibacter*	16S rRNA Sequencing	Chen et al. (2021)
Cirrhosis	↓ *Methanobrevibacter*	16S rRNA Sequencing	Ponziani et al. (2021)

Upward arrows (↑) and downward arrows (↓) represent changes of methanogen abundance and breath-methane excretion, for the respective studies. IBD, Inflammatory bowel disease; CD, Crohn’s Disease; UC, Ulcerative Colitis; IBS, Irritable bowel syndrome; IBS-C, constipation-predominant IBS; IBS-D, diarrhoeal-predominant IBS; SBS, Short Bowel Syndrome; MetS, metabolic syndrome; SIBO, small intestinal bacterial overgrowth. *Denotes detection within respective sample types.

### Methanogenic archaea and gut nutritional ecology

Methanogens occupy a key metabolic niche in anaerobic environments via their utilization of hydrogen gas, as well as the carbon dioxide and/or other simple carbon substrates produced by bacterial fermentation. This process is known as interspecies hydrogen (and carbon dioxide) transfer and serves to limit the build-up of hydrogen gas, which can inhibit bacterial fermentation and growth (Nakamura et al., 2010). The removal of these end products conserves the thermodynamic equilibrium of fermentation, maintaining ‘microbial homeostasis’ within the human GIT (Stams and Plugge, 2009; Sieber et al., 2012). For *Methanosphaera*, a source of methanol is necessary for growth, which can come in the form of free methanol (spirits, beer, wine), methyl esters of fatty acids (aspartame) and pectin (fruit and vegetable) (Toxicity, 2011). There is currently no evidence to suggest *Methanosphaera* can utilize pectin directly, so the degradation of pectin by pectinase-containing bacteria, such as *Bacteroides* spp., is necessary for methanol availability (Jensen and Canale-Parola, 1986; Dongowski et al., 2000). For *Methanomassiliicoccales* spp., methylated-amines produced from dietary carnitine, choline, trimethylamine *N*-oxide (TMAO) and phosphatidylcholine from meat, eggs, nuts, and fish can be utilized. These compounds are broken down by resident microbial communities, such as the conversion of TMAO to trimethylamine (TMA) by *Enterobacteriaceae* spp., to produce free methylated amines (Zeisel et al., 1983; Rebouche and Chenard, 1991; Spencer et al., 2011; Tang et al., 2013; Hoyles et al., 2018). Comparatively, there is little information on the role non-methanogenic archaea play in nutritional ecology of the gut. Given that halophilic archaeal sequences are frequently identified in high salt food products, it is reasonable to assume a portion of halophilic archaeal load may be directly associated with dietary intake (Kobayashi et al., 2000). However, some species of *Halobacteriaceae* are able to survive in salt concentrations similar to that of average salinity levels of healthy individuals (~140 mM sodium) (Fukushima et al., 2007). In addition, small pockets of concentrated luminal ions have also been identified within the colon, potentially acting as favorable micro-niches for these organisms (Naftalin and Pedley, 1995; Spring, 1998). Halophilic archaea have also demonstrated the ability to survive under anaerobic conditions, utilizing electron acceptors such as fumarate for the fermentation of compounds such as arginine (Oxley et al., 2010). Oxley et al. (2010) also noted the increase in luminal osmolality and organic solute concentration of IBD patients as a potential factor for the increase in halophilic archaea. Despite these linkages, the ecological and metabolic niche that halophilic archaea occupy within the GIT is currently inferential, however, with the isolation of human *Haloferax* spp. (Khelaifia and Raoult, 2016; Khelaifia et al., 2017), there is now an opportunity to better define the nutritional ecology of these organisms within the human gut. In fact, a recent study on the bacterial and archaeal composition of colorectal cancer patients showed an increased presence of the halophilic *Natrinema* sp. J7-2 and concurrent reduction in methanogens compared to control subjects (Coker et al., 2020). Additionally, the characterization of the archaeal community of South Korean individuals showed 42.47% archaeal positivity, with 95.54% of archaeal-positive fecal samples containing haloarchaea-associated sequences (Kim J. Y. et al., 2020). Although the average relative abundance of haloarchaea species was 9.63%, some individuals within the cohort displayed a haloarchaea-dominant archaeal community with up to 99.33% relative abundance (Kim J. Y. et al., 2020).

### Methanogenic archaea and gastrointestinal motility

Although there are currently no conclusive findings on the role of methanogenic archaea in human disease, there have been numerous associations made to intestinal-associated pathologies. Breath methane has historically been used to test for the presence of methanogens prior to the development of next-generation sequencing techniques. As summarized by de Lacy Costello et al. (2013), this technique involves the ingestion of a sugar, typically lactose, glucose or fructose, and analysis of alveolar methane over the subsequent 1–2 h period. An increase in the breath methane of constipation-predominant IBS (IBS-C) patients has been associated with increased severity and increased intestinal transit time (Pimentel et al., 2003; Chatterjee et al., 2007). Additionally, an increase in *Methanobrevibacter*, specifically *M. smithii*, has been associated with IBS-C by 16S rRNA sequencing (Pozuelo et al., 2015; Ghoshal et al., 2016). Conversely, individuals with diarrhoeal-predominant IBS show a reduction in both methane production and *Methanobacteriales* abundance (Pimentel et al., 2003; Tap et al., 2017). IBS broadly appears to have no significant association with breath methane or methanogen abundance, though failure to recognize and separate IBS-C/D patients may provide an explanation for these findings (Bratten et al., 2008; Scanlan et al., 2008).

Small intestine bacterial overgrowth (SIBO) is a symptom associated with IBD/IBS patients, in which there is a significant increase in small intestinal bacteria (Colombel et al., 2018). SIBO is relatively common in patients with UC, with ~30% presenting with the condition, compared to a lower prevalence observed in patients with CD (Sandborn, 2009; Lee et al., 2015). A recent study by Suri et al. (2018) showed delayed motility in SIBO to correlate with an increase in breath-methane levels. In a separate study on patients with IBD and SIBO, individuals categorized under IBS-C were more likely to be methane producers compared to IBS-D (58% compared to 28%) (Majewski and McCallum, 2007). Comparatively, individuals with IBS-D were more likely to be hydrogen producers. Given the implication of methanogens and methane in SIBO, recent recommendations by the American College of Gastroenterology include the terminology of intestinal methanogen overgrowth (IMO) to better represent the overgrowth of methanogens in the small intestine and colon (Pimentel et al., 2020). In fact, methane itself has been linked to a reduction in intestinal transit frequency. Jahng et al. (2012) used sections of guinea pig ileum submerged in a peristaltic bath to show an infusion of methane caused decreased peristaltic velocity and increased contraction amplitude, compared to increased peristalsis with hydrogen gas. Additionally, hydrogen was also shown to decrease transit time by 47% in the proximal colon (Jahng et al., 2012). This suggests a possible positive feedback loop between methanogen growth, methane production and increased retention times, caused by a neuromuscular transmitter-like effect of methane (Furnari et al., 2012; Triantafyllou et al., 2014).

Contradictory associations are observed in obese individuals, with an overall increase in the methanogen population but a shift away from *M. smithii* toward unclassified *Methanobrevibacter*, though other studies show no significant association (Zhang et al., 2009; Schwiertz et al., 2010; Million et al., 2012, 2013; Fernandes et al., 2013; Mathur et al., 2013; Maya-Lucas et al., 2018). Individuals with severe malnutrition show reduced *M. smithii* abundance, which may be explained by a lack of intestinal nutrients and thus bacterial fermentation (Million et al., 2016). Indeed, this was recently affirmed in patients with severe acute malnutrition, which showed *M. smithii* in only 4.2% of cases compared to 40.9% in control subjects (Camara et al., 2021). In contrast, individuals with anorexia show a significantly increase in *M. smithii* in multiple studies, as do individuals with metabolic syndrome, suggesting altered microbial communities could affect methanogen populations (Armougom et al., 2009; Mack et al., 2016; Borgo et al., 2017; Lim et al., 2017; Prochazkova et al., 2021).

### Methanogenic archaea and infection?

While methanogens are historically characterized as commensal members of the gut microbiome, recent studies have provided evidence implicating methanogenic archaea in polymicrobial infections. For instance, 16S rRNA gene profiling studies have identified *Methanobrevibacter, Methanobacterium, Methanosarcina, Methanosphaera,* and *Thermoplasmatales* present in subgingival plaque (Belay et al., 1988; Kulik et al., 2001; Robichaux et al., 2003; Li et al., 2009; Horz et al., 2012). However, *M. oralis* is the only species to be significantly associated with periodontal disease, as summarised by Nguyen-Hieu et al. (2013). One of the most common treatment options for periodontitis is metronidazole and is one of the few widely used antibiotics with efficacy against methanogens such as *M. oralis* (Dridi et al., 2011a). Thus, the metronidazole-associated suppression of *M. oralis* may play a significant role in effective treatment (Nguyen-Hieu et al., 2013). Conversely, a separate study showed *M. oralis* to have no significant increase in prevalence for peri-implantitis, suggesting a potential for disease-specific associations in the oral microbiome (Belkacemi et al., 2018). Specific amplification of archaeal 16S rRNA and *mcrA* showed *M. smithii* sequences in chronic paravertebral abscess of a 41-year-old man (Nkamga et al., 2016). The group was also able to isolate *M. oralis* from a nasal sample of a patient suffering from chronic sinusitis. There is also growing evidence that methanogens may contribute to disease progression of brain abscesses. One recent study by Drancourt et al. (2017) showed a higher prevalence of archaeal species by PCR in brain abscess specimens compared with healthy controls. Additionally, metagenomics analysis identified *M. oralis* within multiple abscess samples, as well as several bacterial species, including *Staphylococcus intermedius*. Mice infected cerebrally with *M. oralis, S. intermedius* or both showed significantly increased mortality in all test cases compared to controls. Additionally, co-infection with *M. oralis* and *S. intermedius* showed an increased mortality rate compared to separate infections, suggesting a syntrophic relationship between the microbes. *M. oralis* was further observed in a community-acquired brain abscess of a 30-year-old woman along with *Aggregatibacter actinomycetemcomitans*, again suggesting a potential role for *M. oralis* in infections associated with anaerobic bacteria (Nkamga et al., 2018). These interesting finding could suggest that *Methanobrevibacter* species of the oral or gut microbiome are able migrate from their respective environments and, with bacterial partners, could contribute to the severity of infection. Screening for methanogenic archaea in other “unexpected” regions of the body could further elucidate their role in various disease states, such as tumor samples.

### Interactions between methanogenic archaea and the immune system

Using a murine model of archaeal airway exposure, Blais Lecours et al. (2011) showed that nasal administration of both *M. smithii* and *M. stadtmanae* biomass induced alveolar accumulation of granulocytes and macrophages, as well as thickening of the alveolar septa. While the effects from *M. smithii* challenge were relatively mild, there was a much stronger response toward *M. stadtmanae*, and in a separate study *M. stadtmanae*-induced pneumonitis in mice also caused a significant induction of B-cell-rich tertiary lymphoid tissues (Huppe et al., 2018). When the recruitment of B-cells was prevented by an agonist of sphingosine-1-phosphate receptor 1, a key regulator of lymphoid cells, *M. stadtmanae*-specific lung antibody titres were reduced along with airway leakage and neutrophilic inflammation (Huppe et al., 2018). In a murine model of airway inflammation, crude *Methanosphaera* and *Methanobrevibacter* extracts induced a TH_17_-dependent type IV hypersensitivity response (Bernatchez et al., 2017). Additionally, the *Methanosphaera*-specific immune response also presented with high titres of antigen-specific IgG_1_ and IgG_2a_, again showing the increased immunogenicity of *Methanosphaera* (Bernatchez et al., 2017). Collectively, these results suggest that human archaea, specifically *M. stadtmanae*, stimulate both arms of the immune system and induce a significant proinflammatory immune response. However, further work is needed to understand the archaea-induced inflammatory response in the progression and maintenance of gastrointestinal diseases such as IBD.

Bang et al. (2014) showed that *M. smithii* and *M. stadtmanae* were not recognized by Caco-2/BBe human epithelial cells, in terms of cytokine and antimicrobial peptide production, as was previously shown for intestinal commensal bacteria (Sansonetti, 2004). However, both *Methanosphaera* and *Methanobrevibacter* displayed a decreased growth rate and yield when exposed to a derivative of human cathelicidin, as well as a synthetic anti-lipoprotein peptide (Lpep) and porcine lysin NK-2, when supplemented in axenic culture (Bang et al., 2012). Similarly, *M. luminyensis* showed a high sensitivity to human cathelicidin, though it was significantly more resistant to Lpep and porcine lysin NK-2 (Bang et al., 2017). This mechanism was further explored by identifying specific Toll-like receptors (TLRs) for the recognition of methanogen-specific microbe-associated molecular patterns (MAMPs). Using human embryonic kidney (HEK) cells transfected with specific intracellular TLRs (3, 7, 8, and 9), Bang et al. (2014) showed no activation by RNA or DNA from heat-inactivated archaeal cell preparations. Similarly, no recognition was observed in TLR5 cells, which play an essential role in the recognition of flagella (Bang et al., 2014). This was not unexpected as there is no genetic evidence of flagellin-like genes within *M. stadtmanae* and only two predicted flagellin-like genes within the genome of *M. smithii* PS (Fricke et al., 2006; Samuel et al., 2007). TLR2, NOD1 and NOD2 were also tested for their role in the recognition of bacterial cell membrane components, such as lipid (TLR2) and murein (NOD1/NOD2) (Girardin et al., 2003a,b; Kataoka et al., 2006). Neither TLR2 nor NOD1/NOD2 cells displayed recognition of *M. smithii* and *M. stadtmanae*, suggesting the archaeal cell wall components are immunologically distinct from those of pathogenic bacterial species (Bang et al., 2014). However, contrary to these results, the stimulation of TLR knockout human monocyte BLaER1 cell lines showed not only *M. stadtmanae* itself but also preparations of *M. stadtmanae* RNA to elicit a TLR7- and TL8-specific immune recognition, with the latter showing a greater response (Vierbuchen et al., 2017). Additionally, the TLR8-specific response was able to induce the activation of the NLRP3 inflammasome (Vierbuchen et al., 2017). Thus, further work is warranted to better characterise the potential MAMPs of archaeal species and their associated TLR activation pathway. Despite this variation in response, both strains induced maturation of monocyte-derived dendritic cells (moDCs) through to up-regulation of CD197 and CD86 (Bang et al., 2014). Additionally, confocal and transmission electron microscopy (TEM) was used to show phagocytosis of the methanogens was required for activation of the moDCs (Bang et al., 2014). In a subsequent study, *M. luminyensis* showed a weak response in both moDCs and peripheral blood mononuclear cells (PBMCs), suggesting a lower immunogenic potential toward human immune cells (Bang et al., 2017).

### Methanogenic archaea, IBD, and colorectal cancer

With breath methane testing, individuals with IBD show reduced methane expulsion (McKay et al., 1985; Peled et al., 1987; Gu et al., 2020). Scanlan et al. (2008) further validated these results by PCR of the methanogenesis marker gene *mcrA*. This successfully showed a reduction in the abundance of methanogens in individuals with IBD, with UC patients showing a 24% reduction and patients with CD showing a 30% reduction (Scanlan et al., 2008). Subsequently, patients with CD showed a specific reduction of *Methanobrevibacter*, and a shift toward *Methanosphaera*, which may be responsible for the reduction in breath methane (Blais Lecours et al., 2014; Ghavami et al., 2018). Similarly, this was recently replicated in a population of Kazan IBD patients, which showed a significant reduction of Euryarchaeota, attributed to *Methanobrevibacter*, in patients with CD compared to those with UC (Lo Sasso et al., 2020). Methane metabolism has also been shown to be reduced in patients with IBD compared to control subjects (Nishino et al., 2018). Blais Lecours et al. (2014) specifically showed an increased prevalence of *M. stadtmanae* in patients with IBD compared to control subjects. Additionally, it was also shown that IBD patients produced a significant *M. stadtmanae*-specific IgG immune response compared to non-IBD healthy individuals and PBMCs produced a higher proinflammatory cytokine (TNFα) response when exposed to *M. stadtmanae* compared to *M. smithii*. Stimulation of moDCs also showed *M. stadtmanae* to elicit a significant proinflammatory cytokine response compared to *M. smithii* (Bang et al., 2014).

However, a study on patients with UC and CD from an Indian population showed a converse shift in methanogens, with an increase observed in *Methanobrevibacter* for both patient groups compared to controls (Verma et al., 2010). Individuals with short bowel syndrome (SBS) due to surgical intervention show a decrease in the abundance of *M. smithii*, possibly due to the physical restriction of extended retention times (Boccia et al., 2017). Conversely, individuals with diverticulosis showed an increase in *Methanobrevibacter* compared to standard IBD patients, potentially due to the diverticula creating micro-niches for the methanogens within the colon (Weaver et al., 1986).

Multiple studies show an increase of methanogens and methane production associated with colorectal cancer (CRC) and the stage of disease (Haines et al., 1977; Pique et al., 1984; Segal et al., 1988; Mira-Pascual et al., 2015). However, many studies also show no significant associations between the two, suggesting that the association between methanogens and CRC may involve complex factors that are currently not well understood (Karlin et al., 1982; Hoff et al., 1986; Kashtan et al., 1989; O'Keefe et al., 2007; Scanlan et al., 2008). Although, a recent study on CRC patients showed an enrichment in haloarchaea and a concurrent reduction in methanogens compared to control subjects (Coker et al., 2020).

### Systemic and metabolic disease

*Euryarchaeota* have also been implicated in autoimmune diseases with potential links to the microbiome, such as an increase associated with shorter relapse time for pediatric multiple sclerosis patients (Tremlett et al., 2016a; Castillo-Alvarez et al., 2018) or a correlation to increased disease activity score in patients with rheumatoid arthritis (Picchianti-Diamanti et al., 2018). Recently, Wilmes et al. (2022) and colleagues showed that 2-hydroxypyridine, a metabolite associated with *M. smithii* and methane metabolism, was elevated in patients with Parkinson’s Disease. Further, this compound is a key molecule in disease pathogenesis and may indicate a causal role of *M. smithii* in Parkinson’s Disease. However, recent additional analyses by indicated that *M. smithii* was not the direct source of 2-hydroxypyridine and, as such, further validation is required to determine if *M. smithii* can produce this compound *in vivo* (Wilmes et al., 2023). Adults with asthma were also found to have a reduction in *M. smithii* compared to control subjects (Wang et al., 2018). Individuals with metabolic syndrome (MetS) were observed to have an increase in *Methanobrevibacter* compared to control subjects (Lim et al., 2017).

Despite the correlation of methanogens to various diseases, little information is available on whether methanogens are playing an active role or are simply responding to ecological changes. Some methanogens, namely the *Methanomassiliicoccales,* have been suggested as potential “archaebiotics” for their ability to metabolize TMA (Brugère et al., 2014). TMA is the precursor to uremic toxins TMA oxide (TMAO), which has been associated with cardiovascular diseases, and trimethylaminuria, which causes individuals to emit a pungent fishy odor (Ayesh et al., 1993; Wang et al., 2011; Koeth et al., 2013). Despite demonstrating that cultured isolates of *Methanomassiliicoccales* can metabolise TMA, there are no significant associations observed between these species and atherosclerotic cardiovascular disease (Jie et al., 2017). Similarly, no associations have been observed in patients with chronic kidney disease (CKD), which is also associated with increased levels of uraemic toxins, such as TMAO (Lau et al., 2018). As such, the diseased environment may be unfavorable for these methanogens and further work is required to access the potential of TMA metabolizing methanogens as “archaebiotics”.

### Methodological limitations

Early research of host-associated methanogens was limited by numerous experimental constraints. One such example is the use of bacterial and archaea ‘universal’ primers, which are biased against the majority of archaeal lineages (Raymann et al., 2017). Pausan et al. (2019) demonstrated that a large number of archaeal 16S rRNA-targeting primer pairs demonstrate good coverage *in silico* but fail to detect Thaumarcheota, Woesearchaeota, and only captured a limited diversity of the Euryarchaeota. Additionally, host-associated archaea typically comprise a substantially smaller portion of DNA and contain fewer 16S rRNA gene copy numbers compared to the bacteria (Mahnert et al., 2018). Given their small relative abundance, it is also important to employ effective methods of cell lysis and DNA extraction as typical lysozyme-based lysis methods are ineffective at degrading the cell membranes of archaea (Bang and Schmitz, 2015). Typically, the lack of cultured isolates available for use as reference strains has also been a limitation, however, with the expansion large-scale metagenomic studies, archaeal metagenome-assembled genomes have substantially expanded the number of available representatives from the human gut (Chibani et al., 2022). Nevertheless, representative genomes of low abundance host-associated archaea and those of non-gut origin remain limited. To effectively characterize the host-associated archaeome, archaea-specific DNA extraction methods should be employed, as well as 16S rRNA primer pairs with wider archaeal specificity or metagenomic sequencing of a sufficient depth to detect low abundance archaeal populations. Further, targeted cultivation of novel archaeal lineages would improve reference databases used for culture-independent analyses.

## Future directions in methanogen research

### Unraveling host-specific adaptions to produce selective targets and therapies

Thomas et al. (2022) recently performed a comprehensive study of the abundance and diversity of archaea in the gastrointestinal tracts of 250 animal species (including mammals, birds and fish). They found that the absolute abundance and diversity of archaea in the gut of mammalian species is affected by host phylogeny, diet and intestinal tract physiology, whereas geographical location and host body mass had little effect. Using archaeal specific primers, they detected archaea at the Order- (10) and Family- (19) levels of taxonomy, from 175/250 (70%) of the candidate animal hosts sampled. In line with previous studies, they found the most abundant genera of methanogens to be *Methanobrevibacter*. Amplicons affiliated with *Methanosphaera*, along with *Methanomethylophilaceae*, *Methanocorpusculum,* and *Methanomicrococcus* species were commonly recovered from many samples. These dominant gut methanogens are rarely found in the environment but are closely related to some environmental lineages. This observation suggests that the species of methanogens that inhabit the gut have adapted and specialized to this environment (Thomas et al., 2022). Thomas et al. (2022) also discovered that their amplicons could be assigned to host-specific “clades,” especially for the genus *Methanobrevibacter*, suggesting host adaptation. This study also demonstrated that the abundance of methyl-reducing methanogens, such as *Methanosphaera*, was less affected by dietary fiber content than the hydrogenotrophic methanogens, probably because methanol is largely produced in the gut by the breakdown of pectin. Methyl-reducing methanogens made up around 40% of the total methanogen reads from the animal samples, which differs from environmental samples where this type of methanogenesis is much less common. In that context, the gut environment is likely favorable for this type of methanogenesis, due to the relatively large amounts of choline and pectin present in food and forages, which could have led to the transition from other types of methanogenesis to the methyl-reducing pathway in some gut associated groups, such as *Methanosphaera* spp. and the *Methanomassiliicoccales*. In summary, while Thomas et al. (2022) have proposed there may be several events of adaptation by methanogens to the gut environment, it is difficult to verify these postulates without some whole genome-based and/or biological research with cultured representatives of the major methanogen lineages from different animals hosts.

A study by De La Cuesta-Zuluaga et al. (2021) investigated the adaptations of Methanomassiliicoccales species, which are hydrogen dependent methylotrophs, to the mammalian gut and compared them to other Methanomassiliicoccales that inhabit non-host environments. This study confirmed the existence of two Methanomassiliicoccales clades, one enriched in the gut and one enriched in the environment (with some exceptions). They performed genomic comparisons between these two clades and found that genetic adaptations to the human gut included genome reduction and changes in abundance of genes involved in the shikimate pathway and bile resistance, which is consistent with gut adaptations shown by other methanogens. Gene clusters associated with eukaryote-like proteins and adhesion-like proteins (both are groups of membrane proteins involved in adhesion) were compared, and it was discovered that they were more likely to be found in the host-associated clade and members of the environmental clade enriched in host microbiomes. Adhesion factors are involved in syntrophic relationships with bacterial and eukaryotic organisms, and these results highlight that interaction with the host and other organisms in the microbiome may play an important role in gut adaptation (De La Cuesta-Zuluaga et al., 2021). The environmental clade members were found to have larger genomes and higher gene counts, and the results indicate that the genetic adaptations of Methanomassiliicoccales species differed based on their clade and not their habitat preference. This supports the hypothesis that adaptation to the gut environment occurred separately in each clade, as the host-associated species from each clade have developed different genomic adaptations to this environment.

Despite previous research into the methanogens that reside in the digestive tracts of animals, many host-associated methanogen species are still not well characterized and have not been cultured in isolation. The majority of studies involving gut-associated methanogens have focused on their abundance and ecology, rather than their functional and metabolic diversity. There has been some research into the adaptation of methanogens to the gut environment in general, but very few studies have looked into adaptations by methanogens to specific host animals and the functional diversity that accompanies these adaptations. Further research in this area could lead to a better understanding of their interactions with the host and the microbiome, as well as their roles in the gut environment.

### Methanogens as biomarkers for at risk species

Australia’s unique wildlife are becoming increasingly vulnerable due to anthropogenic factors, such as habitat destruction, agriculture and climate change, and a better understanding of their gut microbiota and nutritional ecology may help with conservation efforts in the future. The microbiome of native herbivores could provide biomarkers for vulnerable animals and give insights into their reactions to anthropogenic pressures. One study by Gibson et al. (2019) showed that, compared to their wild counterparts, captive rhinos had higher abundances of bacterial and archaeal taxa associated with agricultural animals, such as *Methanobrevibacter*, which dominate ruminant archaeal communities. Another study by Yan et al. (2022) demonstrated that *Methanobrevibacter* species were more prevalent in captive versus wild Bactrian Camels, and methanogens on the whole were more abundant in the captive animals. They hypothesize that the wild camels live in harsher environments and, as such, a decrease in methanogenesis in the gut allows for more efficient energy conversion of their food – a critical factor especially when food is scarce. These studies may also indicate that some of the gut microbial taxa found in wild animals were being overtaken by species that are more commonly found in domesticated animals. Similar shifts in gut methanogenic archaea in native Australian mammals may be used as markers to indicate potentially detrimental changes in nutritional and environmental conditions. For this to be effective, an understanding of the methanogens associated with these host animals in the wild and in captivity is required.

The unique diets of some marsupials, their geographical isolation, and the discovery of a non-canonical methanogenesis pathway in *Methanosphaera* sp. WGK6 indicates that it is possible that other, yet uncharacterised, marsupial-associated methanogens could use modified pathways. The discovery of such methanogens would help broaden our current understanding of methanogenesis, as well as possibly aiding our attempts to conserve Australian and global wildlife in the future.

## Concluding remarks

It is evident that methanogenic archaea are prevalent members of host-associated microbiomes, with representatives found in diverse animal hosts from terrestrial and aquatic environments. Although high-throughput metagenomic sequencing has expanded our understanding of the prevalence and abundance of methanogens, there is a lack of cultured representative from the different host-associated species that would allow for functional analysis and validation of cultivation-independent analyses. To understand the role that methanogens play in the host-associated microbiomes of different animal species, it is imperative that diverse methanogen species are cultivated from a variety of animal hosts. More specifically, cultivation attempts should focus on the isolation of novel methylotrophic lineages, such as the *Methanomethylophilaceae*, as well as hydrogenotrophic methanogens other than *Methanobrevibacter*. In addition to characterizing these isolates individually, it is also important to perform *in vitro* and *ex vivo* transcriptomic and proteomic analyses on mixed communities of these methanogens and bacteria from the host animals, such as the methods described by Andersen et al. (2021). These “multi-omics” analyses will provide key information on the wider role of methanogens in the host-associated microbiome and identify strategies for mitigating methane emissions from *Methanobrevibacter*-dominated communities. Further, with the expansions of culture-independent data from the human microbiome, there remains an opportunity to use this data to complement analyses from other mammals and non-human isolates to improve our understanding of host-associated methanogens.

## Author contributions

JV: Conceptualization, Writing – original draft, Writing – review & editing. HM: Writing – original draft, Writing – review & editing. MM: Writing – review & editing.

## Funding

The author(s) declare financial support was received for the research, authorship, and/or publication of this article. The study was supported by an Australian Research Council Discovery Project (DP210103991) awarded to MM. JV was supported by the Research Training Program (RTP) scholarship provided by the Australian Federal Government and the Meat & Livestock Australia post-graduate technical assistance grant (Project code: B.STU.1909). HM is supported by a HDR scholarship from the University of Queensland. MM also acknowledges support of NHMRC CRE Digestive Health via GNT1170893.

## Conflict of interest

The authors declare that the research was conducted in the absence of any commercial or financial relationships that could be construed as a potential conflict of interest.

## Publisher’s note

All claims expressed in this article are solely those of the authors and do not necessarily represent those of their affiliated organizations, or those of the publisher, the editors and the reviewers. Any product that may be evaluated in this article, or claim that may be made by its manufacturer, is not guaranteed or endorsed by the publisher.
